# Compliance with medical recommendations depending on the use of artificial intelligence as a diagnostic method

**DOI:** 10.1186/s12911-021-01596-6

**Published:** 2021-08-06

**Authors:** Michaela Soellner, Joerg Koenigstorfer

**Affiliations:** Chair of Sport and Health Management, Technical University of Munich, Campus D – Uptown Munich, Georg-Brauchle-Ring 60/62, 80992 Munich, Germany

**Keywords:** Artificial intelligence, Diagnostic methods, Compliance

## Abstract

**Background:**

Advanced analytics, such as artificial intelligence (AI), increasingly gain relevance in medicine. However, patients’ responses to the involvement of AI in the care process remains largely unclear. The study aims to explore whether individuals were more likely to follow a recommendation when a physician used AI in the diagnostic process considering a highly (vs. less) severe disease compared to when the physician did not use AI or when AI fully replaced the physician.

**Methods:**

Participants from the USA (n = 452) were randomly assigned to a hypothetical scenario where they imagined that they received a treatment recommendation after a skin cancer diagnosis (high vs. low severity) from a physician, a physician using AI, or an automated AI tool. They then indicated their intention to follow the recommendation. Regression analyses were used to test hypotheses. Beta coefficients (*ß*) describe the nature and strength of relationships between predictors and outcome variables; confidence intervals [*CI*] excluding zero indicate significant mediation effects.

**Results:**

The total effects reveal the inferiority of automated AI (*ß* = .47, *p* = .001 vs. physician; *ß* = .49, *p* = .001 vs. physician using AI). Two pathways increase intention to follow the recommendation. When a physician performs the assessment (vs. automated AI), the perception that the physician is real and present (a concept called social presence) is high, which increases intention to follow the recommendation (*ß* = .22, *95% CI* [.09; 0.39]). When AI performs the assessment (vs. physician only), perceived innovativeness of the method is high, which increases intention to follow the recommendation (*ß* = .15, *95% CI* [− .28; − .04]). When physicians use AI, social presence does not decrease and perceived innovativeness increases.

**Conclusion:**

Pairing AI with a physician in medical diagnosis and treatment in a hypothetical scenario using topical therapy and oral medication as treatment recommendations leads to a higher intention to follow the recommendation than AI on its own. The findings might help develop practice guidelines for cases where AI involvement benefits outweigh risks, such as using AI in pathology and radiology, to enable augmented human intelligence and inform physicians about diagnoses and treatments.

**Supplementary Information:**

The online version contains supplementary material available at 10.1186/s12911-021-01596-6.

## Background

Many recent advancements in digitalization in healthcare build upon the use of big data. Digital technology-enabled big data analyses have the potential to disrupt the healthcare industry as a whole [1–3]. Artificial intelligence (AI) techniques are used in big data analyses to make predictions based on a set of rules. They run calculations from large datasets to estimate different possible solutions for a given problem, and thus enable data-driven decision making [4]. This is why AI might be particularly beneficial in healthcare to help prevent and treat diseases that (1) require learning from large populations; (2) follow patterns that can be detected by technology; and (3) are accessible to physicians and patients.

The advancements in information technology have changed the processes of how diagnoses and treatment recommendations are derived. Algorithms are trained to mimic physicians’ decision-making rules by applying similar rationales when analyzing vast amounts of patient data. AI tools can diagnose certain diseases with expert-level accuracy, and even outperform human experts in some cases [5–9]. More specifically, AI tools can reduce human error, which is particularly beneficial in situations when a patient’s safety is at risk (i.e., for highly [vs. less] severe diseases) [8]. To help patients make the best decision in these situations, they aim to base their decision on state-of-the-art scientific knowledge [10, 11]. Examples of recent uses of AI in this context include the detection of skin and breast cancer as well as of cardiac arrest [6, 12, 13].

Despite the potentially more accurate algorithmic judgment compared to human experts, decision makers are often averse to relying on algorithmic advice [9, 14, 15]. In their evaluation of algorithmic recommendations, as compared to human ones, individuals tend to weigh certain criteria more heavily. In terms of accuracy, algorithmic recommendations are more likely than humans to be rejected when they make mistakes. More specifically, Dietvorst et al. [14] found that even though individuals are aware of the superior performance of algorithms, they are less likely to choose algorithmic advice over human advice. This is because after seeing them both make the same mistake, individuals lose confidence in the algorithm-based advice quicker than in humans [14]. Furthermore, individuals prefer human advice because they subjectively feel that it is easier to understand (despite the importance of accuracy, which might be higher in the case of algorithmic advice) [16]. Individuals may also prefer human advice because it is easier for individuals to shift responsibility to other humans (vs. technology) [9, 17]. Thus, it makes sense that superior performance was found to be an insufficient criterion for creating a preference for algorithms over humans [18]. Longoni et al. [15] documented individuals’ resistance to algorithmic advice across a variety of medical decisions. They showed that individuals’ concern about uniqueness neglect explains the preference for a human provider over an automated provider [15]. Thus, individuals believe that the consideration of the uniqueness of their case is a fundamental human characteristic that an automated provider lacks.

Even though previous studies have described and explained individuals’ aversion to taking advice from algorithms with regard to medical decision making, recent research takes a more nuanced perspective. In several experiments, Logg et al. [19] showed that laypeople readily relied on algorithmic advice (e.g., when making visual estimates and when predicting the popularity of songs). However, this was not uniform across all individuals. Participants with expertise in forecasting were less likely to accept algorithmic advice [19]. Individuals were also found to be more likely to trust and rely on algorithmic recommendations for objective, analytical tasks compared to subjective tasks [16, 18]. There is disagreement in literature about the acceptance of AI in support of physician decision making. Results showed that the combination of human and algorithmic expertise seems to increase the acceptance of AI as long as it does not replace expert judgment [20]. Pezzo and Pezzo [4] found that patients evaluated a physician’s malpractice case more favorably when the physician used a diagnostic decision aid to make the diagnosis compared to a situation in which no such aid was used. In contrast, Shaffer et al. [17] demonstrated that clinicians using a computerized decision aid for their diagnosis were perceived more negatively than physicians making an unaided diagnosis. This is in line with results from Arkes et al. [21] who found that respondents derogated the diagnostic ability of physicians who used a computer-based decision support.

With few exceptions [12], there is little data on the general mechanism that affects whether patients comply with recommendations depending on the use of AI technology and the level of human involvement in the medical assessment. Furthermore, previous studies solely compared two conditions: physician vs. AI or physician vs. physician supported by AI. To our knowledge there is so far no study that contrasted all three forms of diagnostic methods in one study. The present study aimed to partially fill this gap. The study sought to explore whether individuals were more likely to follow a recommendation when a physician used AI to derive a diagnosis and give a treatment recommendation as compared to situations in which the physician did not use any AI or when AI fully replaced the physician. The study also considered the severity of the disease that AI might give diagnostic and treatment recommendations for as well as two mediating variables: social presence and innovativeness.

### The roles of social presence and innovativeness

The question of whether an individual complies with a medical recommendation is determined by both socioemotional and functional elements of a medical assessment [10, 22, 23]. Social presence refers to the extent to which an individual perceives that a social element is present [24]. Social presence theory postulates that different communication media can be classified according to their social presence—an indicator of the degree of awareness of another person within the interaction—and that social presence influences the quality of the interaction [25]. High social presence creates the sense of a personal, sociable, and sensitive being [26]. This is not limited to interactions between human beings, but can also include computer-mediated communication [27]. Based on the reciprocal nature of a patient’s interaction with a physician, social presence should be higher when a human agent (with or without the aid of technology) provides the medical service as compared to an automated provider. Social presence was found to positively affect health outcomes [28]. Thus, perception of someone being present or taking care of the patient can then be assumed to positively influence an individual’s intention to comply with a medical recommendation.

The evaluation of the functional performance of the medical assessment is another determinant of compliance with a medical recommendation. The use of technology in the medical assessment might indicate to patients that state-of-the-art technology is being utilized and can serve as an indicator for the level of innovativeness in the delivery of the medical service [29]. Innovativeness serves as a proxy for newness and, in the medical field, for the consideration of the latest scientific knowledge. Individuals associate innovative technology with success and advancement [30]. This association prompts individuals to evaluate new technology performance favorably [31]. An individual’s perception of the innovativeness of a diagnostic method should be higher when AI is used because AI can apply recent scientific knowledge with the support of information technology and reliance on big healthcare data (with or without the involvement of physicians). An individual’s intention to comply with a recommendation might increase because he or she may feel that the recommendation is based on state-of-the-art scientific evidence. The downstream effects of both social presence and perceived innovativeness are postulated to affect an individual’s willingness to comply with a medical recommendation for the treatment.

The aim of this study was to explore whether the utilization of AI technologies in the medical assessment process influenced patient compliance intention with a recommended treatment. Based on this, we posed two research questions. First, we wanted to explore whether there were differences in an individual’s intention to follow a medical recommendation depending on the diagnostic method used (physician, physician using AI, or automated AI tool alone) and the level of disease severity (high or low). Second, the study aimed to explore how individuals perceive the three diagnostic methods with regard to their social presence and innovativeness, as well as whether social presence and perceived innovativeness influence an individual’s intention to comply with the treatment recommendation.

## Methods

### Study design, procedure, and sample

To answer the research questions, we used a 3 (diagnostic method: physician vs. physician who uses AI vs. automated AI tool) by 2 (severity of the disease: high vs. low) experimental design. Participants were randomly assigned to one of the six experimental conditions.

The study was conducted online, and participants were recruited through Amazon Mechanical Turk, an online platform via which registered individuals complete tasks in return for small payments. The use of Amazon Mechanical Turk workers is appropriate given the design of the study (use of vignettes and a randomized assignment to experimental groups; see [15, 32–34] on the use of the population to study medicine- and health-related topics). We limited our sample to individuals who were 18 years of age or older and located in the USA.

Prior to starting the study, informed consent was obtained and the study procedure was explained to the participants. During the study, the participants first read a hypothetical scenario and then filled in a survey. Participants were fully debriefed at the end of the study. The study was conducted in accordance with the 1964 Helsinki declaration and its later amendments. The Faculty Board of the TUM Germany, which acts as the local ethics committee for studies outside the TUM Faculty of Medicine, approved the study.

### Decision scenarios

Participants first read a scenario. Six different versions of the scenario on the detection and treatment of skin cancer were developed, manipulating the diagnostic method and the severity of the disease. The scenario was kept similar in all other respects.

**Fig. 1:**
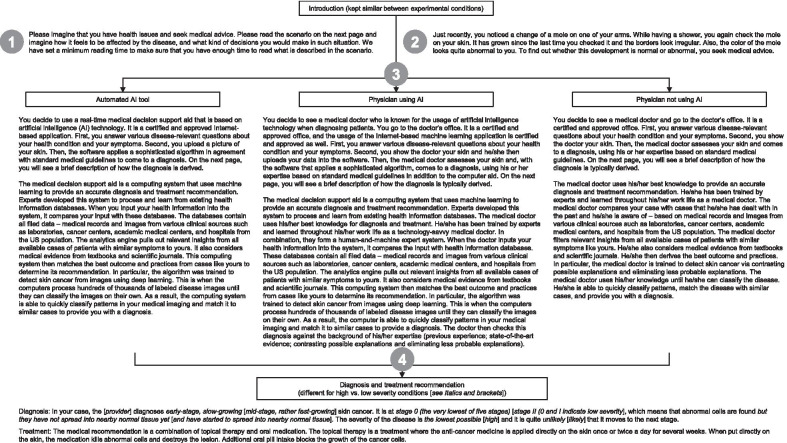
Vignettes for the sole use of the automated AI tool vs. physician using AI vs. physician not using AI as the diagnostic method

In the scenario, participants were asked to imagine that they recently noticed a change in a mole on their skin and that they decided to seek medical advice. The scenario then described a visit to a healthcare provider, where the medical assessment was performed either by a physician, a physician using AI technology, or an automated AI tool alone (described as a real-time medical decision support aid based on AI technology). Further information was provided to the participants regarding how the respective method works to generate the diagnosis and treatment recommendation. Subsequently, half of the participants were told that the diagnosis of their disease (skin cancer) was not severe (stage 0) and the other half was told that it was severe (stage 2). Participants then read that they received a medical recommendation for the treatment, which was identical for each condition (i.e., topical therapy and oral medication). After receiving the recommendation, participants indicated their intention to comply with it. Figure 1 shows the vignettes that the participants read.

### Measures

The questionnaire that was used in the study can be downloaded online (Additional file 1). The intention to comply with the recommendation was measured via six items, rated on a seven-point rating scale. Amongst others, items included the following: “How likely would you follow the medical recommendation?”, anchored at 1 = very unlikely and 7 = very likely; “What is the probability that you will stick to the recommendation?”, anchored at 1 = very improbable and 7 = very probable. Internal consistency, as calculated by Cronbach’s alpha, was high (*α* = 0.89).

Social presence measures were obtained from Gefen and Straub [35] and adapted to the context of the study. The items included the following: “There is a sense of personalness in the process”; “There is a sense of sociability in the process”; and “There is a sense of human warmth in the process.” Participants rated their responses on a seven-point rating scale (1 = strongly disagree, 7 = strongly agree; *α* = 0.96).

Perceived innovativeness of the diagnostic method was measured via three pre-tested items. Participants indicated their level of agreement to the following three statements: “There is a sense of technological advancement in the process”; “There is a sense of innovativeness in the process”; and “There is a sense of state-of-the-art knowledge generation in the process.” Participants rated their responses on a seven-point rating scale (1 = strongly disagree, 7 = strongly agree; *α* = 0.84). Mean scores were calculated for each latent variable.

### Statistical analysis plan

Analyses were conducted using SPSS 25. We examined descriptive statistics for the sample. We then conducted multiple linear regression analyses to test whether the two predictors (diagnostic method and disease severity) influenced the intention to comply with the medical recommendation, as well as social presence and innovativeness. For the analyses, we created two dummies for the diagnostic method variable. The coding for the respective dummies was as follows: dummy one: physician = 1, other conditions = 0; dummy two: physician using AI = 1, other conditions = 0. The automated provider serves as baseline for comparison in the analysis. Disease severity was coded as 0 = not severe and 1 = severe.

**Fig. 2:**
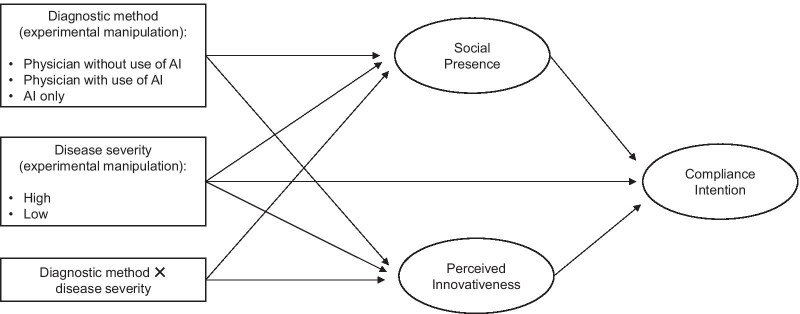
Conceptual model of the influence of the type of diagnostic method, the disease severity, and their interactions on intention to comply with the recommendation via social presence and perceived innovativeness of the diagnostic method

We also examined whether social presence and innovativeness mediated the relationship between the diagnostic method and the intention to comply with the medical recommendation depending on the severity of the disease. We used Hayes’ [36] PROCESS model 4 (version 3 for SPSS), including bootstrapping procedures. This is an approach that permits simultaneous testing of the direct, indirect, and total effects of the type of diagnostic method, disease severity and interactions on the intention to follow the medical recommendation through the two parallel mediators. Two diagnostic method dummy variables [37] were modeled as predictors; disease severity and the interactions of the two variables were included as covariates in the model. The dependent variable was the intention to comply with the recommendation (Fig. 2). Beta coefficients (*ß*, unstandardized) describe the degree of change in the dependent variable for a one-unit change in the predictor variable. Confidence intervals [*CI*] that exclude zero indicate significant mediation effects.

## Results

### Sample characteristics

Four hundred fifty-two individuals, 196 of them females (43.8%), participated in the study. All were located in the USA, with a mean age of 41.7 years (*SD* = 15.6) (see Table 1). The World Health Organization [38] reports that one in every three cancers diagnosed is a form of skin cancer, and according to statistics from the Skin Cancer Foundation, one in every five Americans will develop skin cancer in their lifetime. In our sample, 12.1% participants had previously been diagnosed with skin cancer, 28.8% of them had a previous skin cancer diagnosis in their family, while the remaining 59.2% did not have any skin cancer history.

**Table 1:** Sample characteristics

Variable	Percentage of participants
Age	
18–24 years	6.0
25–34 years	37.5
35–44 years	13.9
45–54 years	12.0
55 years and older	30.6
Education	
Less than high school diploma	0.7
High school diploma	9.6
Some college or associates degree	20.1
Bachelor’s degree	46.4
Master’s degree	21.2
Professional degree	1.1
Doctorate	0.9

### Differences in intentions and mediators depending on the diagnostic method and disease severity

**Fig. 3:**
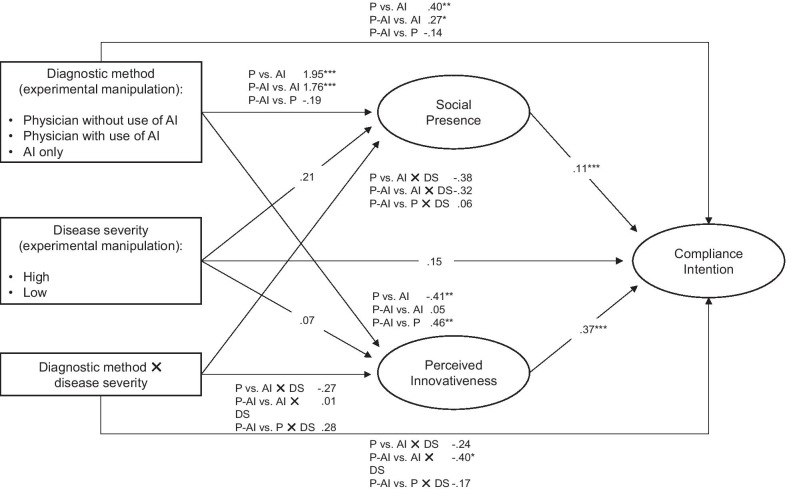
Results of the mediation model. *Notes*. To obtain coefficients for the comparison of physician plus AI and physician, we recoded dummy two with physician as reference group. The figure reports unstandardized beta coefficients. *p < 0.05, **p < 0.01, ***p < 0.001. Abbreviations: P = Physician without use of AI, P-AI = Physician using AI, AI = Automated AI tool, DS = Disease severity

The model explains 23.4% of the variance in the intention to comply with the medical recommendation (see Fig. 3 for the path coefficients). Compared to the automated AI tool alone, the intention to comply with the medical recommendation was significantly greater for the physician (*ß* = 0.40, *SE* = 0.14, *p* = 0.005) and physician using AI (*ß* = 0.27, *SE* = 0.14, *p* = 0.05; see Table 2) variables. The difference between the physician using AI and the physician variable was non-significant (*ß* = − 0.14, *SE* = 0*.*13,* p* = 0.30).

**Table 2:** Relative direct and indirect effects of the predictors on an individual’s intention to comply with a medical recommendation

Effects	*ß*	*SE*	*p [95% CI]*
*Direct effects on the mediators*			
Constant	3.66	0.16	< 0.001
Physician → social presence	1.95	0.23	< 0.001
Physician plus AI → social presence	1.76	0.23	< 0.001
Disease severity → social presence	0.21	0.23	0.35
Physician × disease severity → social presence	− 0.32	0.32	0.32
Physician plus AI × disease severity → social presence	− 0.38	0.32	0.24
Constant	5.98	0.11	< 0.001
Physician → perceived innovativeness	− 0.41	0.15	0.002
Physician plus AI → perceived innovativeness	0.05	0.15	0.73
Disease severity → perceived innovativeness	0.07	0.15	0.66
Physician × disease severity → perceived innovativeness	− 0.27	0.21	0.20
Physician plus AI × disease severity → perceived innovativeness	0.01	0.21	0.98
*Direct effects on the dependent variable*			
Constant	2.96	0.27	< 0.001
Physician → compliance intention	0.40	0.14	0.005
Physician plus AI → compliance intention	0.27	0.14	0.05
Social presence → compliance intention	0.11	0.03	< 0.001
Perceived innovativeness → compliance intention	0.37	0.04	< 0.001
Disease severity → compliance intention	0.15	0.13	0.24
Physician × disease severity → compliance intention	− 0.24	0.19	0.20
Physician plus AI × disease severity → compliance intention	− 0.40	0.19	0.03
*Indirect effects *via* the mediators*			
Physician, total indirect effect	0.07	[− 0.12, 0.29]	
Via social presence	0.22	[0.09, 0.39]	
Via perceived innovativeness	− 0.15	[− 0.28, − 0.04]	
Physician plus AI, total indirect	0.22	[0.03, 0.41]	
Via social presence	0.20	[0.07, 0.36]	
Via perceived innovativeness	0.02	[− 0.09, 0.12]	

The experimental group that was assigned to the automated AI tool as the diagnostic method served as the reference group for comparison with dummy one (physician) and dummy two (physician plus AI)

*ß* = Unstandardized path coefficient, *SE* = Standard error, *p* = Significance, *CI* = Confidence interval

The effect of the severity of the disease on the intention to comply with the medical recommendation was non-significant (*ß* = 0.15, *SE* = 0 0.13, *p* = 0.24). The analysis revealed one significant interaction effect between the physician using AI (vs. the automated AI tool alone) and the severity of the disease (*ß* = − 0.40, *SE* = 0.19, *p* = 0.03). The participants’ intention to follow the medical recommendation was greater in the scenario of the physician using AI compared to the automated AI tool when the severity of the disease was low (but not when it was high).

The effects of both social presence (*ß* = 0.11, *SE* = 0.28, *p* < 0.001) and perceived innovativeness (*ß* = 0.37, *SE* = 0.04, *p* < 0.001) on the intention to comply with the medical recommendation were significant. Furthermore, social presence was higher when a physician (*ß* = 1.95, *SE* = 0.23, *p* < 0.001) or a physician using AI (*ß* = 1.76, *SE* = 0.23, *p* < 0.001) made the assessment, compared to the automated AI tool alone. The effect on social presence was not significantly different between the physician and the physician using AI.

Perceived innovativeness was higher when a physician using AI (*ß* = 0.46, *SE* = 0.15, *p* = 0.002) or when the automated AI tool (*ß* = − 0.41, *SE* = 0.15, *p* = 0.01) made the assessment, compared to the physician not using AI. The effect on perceived innovativeness was not significantly different for the comparison between the automated AI tool and the physician using AI.

The severity of the disease had no significant effect on social presence (*ß* = 0.21, *SE* = 0.23, *p* = 0.35) or perceived innovativeness (*ß* = 0.07, *SE* = 0.15, *p* = 0.66). All interaction effects between the diagnostic method and the disease severity on the two mediators were non-significant.

Next, we calculated the indirect effects of the type of diagnostic method on an individual’s intention to comply with the medical recommendation. For the comparison between the physician and the automated AI tool, both social presence (*ß* = 0.22, 95% confidence interval (*CI*) [0.09, 0.39]) and perceived innovativeness (*ß* = − 0.15, *95% CI* [− 0.28, − 0.04]) were significant mediators (the *CI*s excludes zero). For the comparison between the physician using AI and the automated AI tool, social presence (*ß* = 0.20, *95% CI* [0.07, 0.36]), but not perceived innovativeness (*ß* = 0.02, *95% CI* [− 0.09, 0.12]), was a significant mediator. When comparing the physician using AI with the physician alone, there was a significant indirect effect via perceived innovativeness (*ß* = 0.17, *95% CI* [0.06, 0.30]) but not via social presence (*ß* = − 0.02, *95% CI* [− 0.01, 0.07]).

## Discussion

The purpose of the study was to explore whether individuals were more likely to follow a medical recommendation when a physician used AI to derive a diagnosis and recommend a treatment compared to situations in which AI was not used or in which the physician was completely replaced by AI. The results of the experimental study, which used skin cancer as the case, showed that intention to follow the medical recommendation was greater for the physician compared to the automated AI tool alone (regardless of disease severity) and for the physician using AI compared to the automated AI tool alone (in the case of low disease severity). There was no difference between the physician using AI and the physician alone. The results thus indicate that individuals were most likely to stick to the recommendations, a factor that should increase recovery and health outcomes [39], when human expertise was central to the diagnosis and the treatment recommendation (vs. an automated AI tool). This finding is in agreement with a literature review on AI innovations in healthcare, which showed that AI is best used to supplement human expertise, potentially benefitting clinical skills and enriching patient-physician interactions [40; see also 41 for an earlier review].

In the present study, intention to follow the medical recommendation was greater in the case of the physician using AI as compared to the automated AI tool alone when the severity of the disease was low (but not when it was high). Thus, when AI was included in a physician’s efforts, the treatment recommendations for less severe diseases were more likely to be accepted as compared to recommendations made by an automated AI tool alone (which most of the individuals resisted adhering to). When only humans were involved in the medical assessment, social presence was high and the innovativeness perception was low (vs. use of an automated AI tool), which influenced intended compliance. When only AI is used for an automated assessment, innovativeness perception was high but social presence was low (vs. when a physician was involved), which also influenced intended compliance. There was no significant difference between the physician using AI and the automated AI tool alone in terms of perceived innovativeness, and it did not impair social presence.

The study makes three important contributions to the medical decision-making literature. First, it extends social presence theory by proposing two pathways for an individual’s compliance with treatment recommendations via social presence and perceived innovativeness. Previous studies have looked at social presence as the sole mediator, even though researchers have criticized that the concept falls short with regard to the technology-enabling characteristics of the interaction, such as an agent’s capability to be up-to-date, innovative, and act according to the state-of-the-art of knowledge [42]. The present study partially fills this research void. The pathways have different magnitudes depending on the diagnostic method under consideration (physician vs. physician using AI vs. automated AI tool alone). The involvement of humans and the involvement of technology in the medical assessment may operate via different mechanisms when individuals form compliance intentions. These findings are of interest to compliance research, indicating that AI tools may need a high degree of innovativeness in order to increase compliance intentions [4].

Second, participants’ adherence intentions were higher when hypothetically diagnosed with a disease relatively low in seriousness, by a physician using AI (vs. automated AI), compared to a more serious condition. This is in line with previous findings that those who are worse in health are less likely to be adherent [43, 44]. A potential explanation is that many physical, psychological and practical limitations disrupt patients’ adherence efforts [43]. Patients may have doubts about the efficacy of their treatments [45], their expectations for and interactions with their provider may be reduced in quality as they grow more severely ill [46], or they may become hopeless or depressed [47].

Finally, the study provides evidence for the general notion that a human–technology combination (here, physicians’ use of AI to diagnose a disease) may lead to compliance intentions that are as high as with physician-only consults. Most importantly, for the case of skin cancer considered here, the sole use of AI technology as a diagnostic method might negatively affect the intent to comply with the recommendation treating the disease. In general, patients are skeptical about using a computer-generated algorithm for decision making [14]. Contrary to previous findings [21], our results have positive implications for healthcare service providers considering the implementation of AI tools. The sole use of AI as a diagnostic method cannot be recommended based on the results of the present study. Still, as a means to promote the perception of innovativeness, physicians might use AI tools as a diagnostic aid. The usefulness of combining physicians’ expertise with the use of AI tools has been shown by Han et al. [48], who used convolutional neural network architectures to both diagnose skin cancer and select treatment options. They found maximal effectiveness when the deep learning algorithm acts as an “augmented intelligence” [p. 6] aid. Thus, the findings of the present study in combination with AI research in healthcare might help develop practice guidelines for cases where AI involvement benefits outweigh risks, such as using AI in pathology and radiology, to enable augmented human intelligence and inform physicians about diagnoses and treatments. Physicians therefore have the option to integrate and utilize suitable hardware and software that combine the expertise of AI technology with the physician’s expertise [49].

This study has several limitations. First, participants were not able to choose their preferred diagnostic method. They were randomly assigned to one of the three methods. A lack of trust in the method could have had a negative effect on individuals’ compliance intentions. Also, there might be differences in the degree of familiarity with the various diagnostic methods based on previous personal experiences. While individuals might be more familiar with the medical service provided by a physician, they might be less familiar with the use of AI in healthcare. Although it is impossible to rule out the influence of these past learnings in empirical studies, future research could use videos (e.g., best practice examples, instructions) or trials to educate individuals and then assess the influence of trust and habits on the acceptance of and compliance with AI-derived or AI-supported medical decisions.

Second, instead of manipulating the severity of the disease, future research might manipulate the complexity of the disease (given AI’s ability to solve complex problems). If patients are informed about the high (vs. low) complexity of diseases, they might be more open towards AI-derived or AI-supported medical decisions. In the present study, the type of disease was kept constant to rule out alternative explanations (implying that similar processes produce skin cancer cells). Future research might use different diseases to manipulate the complexity or use vignettes with different descriptions of the complexity of a disease.

Third, individuals’ perceptions may vary depending upon the specific type of decision scenario. For example, decisions that are more (vs. less) value-laden, preference-laden, or rather considered routine problems (e.g., upper respiratory infection vs. cancer) might make patients less likely to accept AI. Future research might test the hypothesis that AI might not perform well on value- or preference-laden decisions (which often require shared decision making) compared to others that are not (e.g., diagnosis which is straightforward and for which there is one well-established standard of care). The current study only assessed one type of scenario.

Fourth, the study does not separate out use of AI to diagnose versus treat conditions. However, individuals’ perceptions may be different for using AI to diagnose versus make treatment decisions. For example, AI might help a provider to diagnose the condition (e.g., in the area of radiology or pathology) but the provider alone might make the decision for what treatment to use. Hence, future research might be devoted to finding out peculiarities in effects depending on AI’s involvement at different stages of patient-provider interactions. Furthermore, the scenario used a specific treatment recommendation: topical therapy and oral medication. Yet, the severity of the treatment could influence individuals’ intentions to comply with the recommendation. They may be more willing to adhere to topical therapy and oral medication compared to surgery, radiation therapy, or chemotherapy. Future research might assess whether, and how, the perceived severity of the treatment interacts with the involvement of AI.

Lastly, it was not real behavior but only behavioral intentions that were assessed in the studies. Future studies might consider patients who have been diagnosed with the disease and who are willing to report their actual compliance-related behavior (e.g., intake of medicine, lifestyle behavior changes). Randomized control trials would be needed to assess cause-effect relationships between different providers and actual compliance outcomes. Also, in the present study, the scenarios did not mention any particular aspects of human warmth (e.g., conversations, empathy from physicians), thereby restraining the context for social presence. Future studies might also focus on the reciprocal interaction between patients and healthcare service providers. This might increase the external validity of the findings.

## Conclusion

AI applications in healthcare are getting increasingly pervasive. The findings of this study showed that the level of AI involvement in the diagnostic process determines patients’ compliance intentions with medical recommendations. The replacement of humans in the medical assessment by automated AI tools reduces compliance intentions compared to the combination of human and AI-based technological expertise. Both social presence and innovativeness are important factors that drive intended adherence with medical recommendations.

## Supplementary Information

**Additional file 1**: Questionnaire.

## Data Availability

Datasets used and analyzed during this current study are available from the corresponding author upon reasonable request.

## Abbreviations

AI: Artificial intelligence
P: Physician without use of AI
P-AI: Physician using AI
AI: Automated AI tool
DS: Disease severity

## References

[1] Agarwal R, Gao G, DesRoches C, Jha AK (2010). The digital transformation of healthcare: current status and the road ahead. Inform Syst Res.

[2] Günther WA, Rezazade Mehrizi MH, Huysman M, Feldberg F (2017). Debating big data: a literature review on realizing value from big data. J Strategic Inf Syst.

[3] Wang Y, Hajli N (2016). Exploring the path to big data analytics success in healthcare. J Bus Res.

[4] Pezzo MV, Pezzo SP (2006). Physician evaluation after medical errors: does having a computer decision aid help or hurt in hindsight?. Med Decis Making.

[5] Haenssle HA, Fink C, Schneiderbauer R, Toberer F, Buhl T, Blum A, Kalloo A, Hassen ABH, Thomas L, Enk A, Uhlmann L (2018). Man against machine: diagnostic performance of a deep learning convolutional neural network for dermoscopic melanoma recognition in comparison to 58 dermatologists. Ann Oncol.

[6] Abramoff MD, Lavin PT, Birch M, Shah N, Folk JC (2018). Pivotal trial of an autonomous AI-based diagnostic system for detection of diabetic retinopathy in primary care offices. npj Digital Med.

[7] Beck AH, Sangoi AR, Leung S, Marinelli RJ, Nielsen TO, van de Vijver MJ, Koller D (2011). Systematic analysis of breast cancer morphology uncovers stromal features associated with survival. Sci Transl Med.

[8] Balas AE (2001). Information systems can prevent errors and improve quality. J Am Med Inform Assn.

[9] Promberger M, Baron J (2006). Do patients trust computers?. J Behav Decis Making.

[10] Gino F, Moore DA (2007). Effects of task difficulty on use of advice. J Behav Decis Making.

[11] Bertsimas D, Orfanoudaki A, Weiner RB (2020). Personalized treatment for coronary artery disease patients: a machine learning approach. Health Care Manag Sci.

[12] Marr B. How is AI used in healthcare—5 powerful real-world examples that show the latest advances. https://www.forbes.com/sites/bernardmarr/2018/07/27/how-is-ai-used-in-healthcare-5-powerful-real-world-examples-that-show-the-latest-advances/#197bfff05dfb (2018). Accessed 03 Dec 2018.

[13] Supriya M, Deepa AJ (2020). A novel approach for breast cancer prediction using optimized ANN classifier based on big data environment. Health Care Manag Sci.

[14] Dietvorst BJ, Simmons JP, Massey C (2015). Algorithm aversion: people erroneously avoid algorithms after seeing them err. J Exp Psychol Gen.

[15] Longoni C, Bonezzi A, Morewedge C (2019). Resistance to medical artificial intelligence. J Consum Res.

[16] Yeomans M, Shah A, Mullainathan S, Kleinberg J (2019). Making sense of recommendations. J Behav Decis Making.

[17] Shaffer VA, Probst CA, Merkle EC, Arkes HR, Medow MA (2013). Why do patients derogate physicians who use a computer-based diagnostic support system?. Med Decis Making.

[18] Castelo N, Bos MW, Lehmann DR (2019). Task-dependent algorithm aversion. J Marketing Res.

[19] Logg JM, Minson JA, Moore DA (2019). Algorithm appreciation: people prefer algorithmic to human judgment. Organ Behav Hum Dec Process.

[20] Palmeira M, Spassova G (2015). Consumer reactions to professionals who use decision aids. Eur J Marketing.

[21] Arkes HR, Shaffer VA, Medow MA (2007). Patients derogate physicians who use a computer-assisted diagnostic aid. Med Decis Making.

[22] White TB (2005). Consumer trust and advice acceptance: the moderating roles of benevolence, expertise, and negative emotions. J Consum Psychol.

[23] Wirtz J, Patterson PG, Kunz WH, Gruber T, Lu VN, Paluch S, Martins A (2018). Brave new world: service robots in the frontline. J Serv Manage.

[24] Short J, Williams E, Christie B (1976). The social psychology of telecommunications.

[25] Xin C, Youjia F, Barbara L (2015). Integrative review of social presence in distance education: issues and challenges. Educ Res Rev.

[26] Lankton NK, McKnight DH, Tripp J (2015). Technology, humanness, and trust: rethinking trust in technology. J Assoc Inf Syst.

[27] Biocca F, Harms C, Burgoon JK (2003). Towards a more robust theory and measure of social presence: review and suggested criteria. Presence Teleoperators Virtual Environ.

[28] Sambo CF, Howard M, Kopelman M, Williams S, Fotopoulou A (2010). Knowing you care: effects of perceived empathy and attachment style on pain perception. Pain.

[29] Fichman RG, Dos Santos BL, Zheng ZE (2014). Digital innovation as a fundamental and powerful concept in the information systems curriculum. MIS Quart.

[30] Elsbach KD, Stigliani I (2019). New information technology and implicit bias. Acad Manage Perspect.

[31] Clark BB, Robert C, Hampton SA (2016). The technology effect: how perceptions of technology drive excessive optimism. J Bus Psychol.

[32] Ellis EM, Klein WMP, Orehek E, Ferrer RA (2018). Effects of emotion on medical decisions involving tradeoffs. Med Decis Making.

[33] Hopkin G, Au A, Collier VJ, Yudkin JS, Basu S, Naci H (2019). Combining multiple treatment comparisons with personalized patient preferences: a randomized trial of an interactive platform for statin treatment selection. Med Decis Mak.

[34] Manigault AW, Handley IM, Whillock SR (2015). Assessment of unconscious decision aids applied to complex patient-centered medical decisions. J Med Internet Res.

[35] Gefen D, Straub DW (2004). Consumer trust in B2C e-commerce and the importance of social presence: experiments in e-products and e-services. Omega.

[36] Hayes AF (2013). Introduction to mediation, moderation, and conditional process analysis: a regression-based approach.

[37] Spiller SA, Fitzsimons GJ, Lynch JG, McClelland GH (2013). Spotlights, floodlights, and the magic number zero: simple effects tests in moderated regression. J Marketing Res.

[38] World Health Organization (2019) How common is skin cancer? https://www.who.int/uv/faq/skincancer/en/index1.html

[39] Bonaccio S, Dalal RS (2006). Advice taking and decision-making: an integrative literature review, and implications for the organizational sciences. Organ Behav Hum.

[40] Salla E, Pikkarainen M, Leväsluoto J, Blackbright H. AI innovations and their impact on healthcare and medical expertise. In: Bitran I, Conn S, Huizingh KRE, Torkeli M, Tynnhammar M, editors. ISPIM innovation conference proceedings; 2018.

[41] Garg AX, Adhikari NKJ, McDonald H, Rosas-Arellano MP, Devereaux PJ, Beyene J, Sam J, Haynes RB (2005). Effects of computerized clinical decision support systems on practitioner performance and patient outcomes: a systematic review. JAMA.

[42] Lu B, Fan W, Zhou M (2016). Social presence, trust, and social commerce purchase intention: an empirical research. Comput Hum Behav.

[43] DiMatteo MR, Haskard KB, Williams SL (2007). Health beliefs, disease severity, and patient adherence: a meta-analysis. Med Care.

[44] Christensen H, Griffiths KM, Farrer L (2009). Adherence in internet interventions for anxiety and depression. J Med Internet Res.

[45] Horne R, Weinman J (1999). Patients’ belief about prescribed medicine and their role in adherence to treatment in chronic physical illness. J Psychosom Res.

[46] Kravitz R, Bell RA, Azari R, Krupat E, Kelly-Reif S, Thorn D (2002). Request fulfillment in office practice: antecedents and relationship to outcomes. Med Care.

[47] Carney RM, Freedland KE, Eisen SA, Rich MW, Jaffe AS (1995). Major depression and medical adherence in elderly patients with coronary artery disease. Health Psychol.

[48] Han SS, Park I, Chang SE, Lim W, Kim SM, Park GH, Chae JB, Huh CH, Na JI (2020). Augmented intelligence dermatology: deep neural networks empower medical professionals in diagnosing skin cancer and predicting treatment options for 134 skin disorders. J Invest Dermatol.

[49] Tschandl P, Rinner C, Apalla Z, Argenziano G, Codella N, Halpern A, Janda M, Lallas A, Longo C, Malvehy J, Paoli J, Puuig S, Rosendahl C, Soyer HP, Zalaudek I, Kittler H (2020). Human–computer collaboration for skin cancer recognition. Nature Med.

