# Supplementary material for: Compliance with medical recommendations depending on the use of artificial intelligence as a diagnostic method
Source: BMC Med Inform Decis Mak. 2021 Aug 6;21:236. doi: 10.1186/s12911-021-01596-6 (PMC8344186; doi:10.1186/s12911-021-01596-6)
Supplement: Supplementary file 1 — Additional file 1: Questionnaire. [file 12911_2021_1596_MOESM1_ESM.docx]

**Questionnaire**

| Intention | 1 | Please indicate how likely you would follow the medical recommendation of the [*provider*^a^] | 1 = very unlikely; 7 = very likely |
| --- | --- | --- | --- |
|  | 2 | What is the probability that you will stick to the recommendation of the [*provider*]? | 1 = very improbable; 7 = very probable |
|  | 3 | How closely would you follow the recommendation of the [*provider*]? | 1 = not closely at all; 7 = very closely |
|  | 4 | How likely will there be a positive outcome when sticking to the recommendation? | 1 = very unlikely; 7 = very likely |
|  | 5 | How certain is it that you would achieve positive outcomes when sticking to the recommendation? | 1 = very uncertain; 7 = very certain |
|  | 6 | What is the probability of having positive outcomes when you follow the recommendation? | 1 = very improbable; 7 = very probable |
| Social Presence | 1 | There is a sense of human warmth in the process | 1 = strongly disagree; 7 = strongly agree |
|  | 2 | There is a sense of sociability in the process | 1 = strongly disagree; 7 = strongly agree |
|  | 3 | There is a sense of personalness in the process | 1 = strongly disagree; 7 = strongly agree |
|  | 4^b^ | There is a sense of human sensitivity in the process | 1 = strongly disagree; 7 = strongly agree |
|  | 5^b^ | There is a sense of human contact in the process | 1 = strongly disagree; 7 = strongly agree |
| Innovativeness | 1 | There is a sense of technological advancement in the process | 1 = strongly disagree; 7 = strongly agree |
|  | 2 | There is a sense of innovativeness in the process | 1 = strongly disagree; 7 = strongly agree |
|  | 3 | There is a sense of state-of-the-art knowledge generation in the process | 1 = strongly disagree; 7 = strongly agree |
| Agreeableness | 1 | Following the recommendation would improve my well-being | 1 = strongly disagree; 7 = strongly agree |
|  | 2 | Following the recommendation would improve my health | 1 = strongly disagree; 7 = strongly agree |
|  | 3 | I would be well off if I followed the recommendation | 1 = strongly disagree; 7 = strongly agree |
| Trust | 1 | How much do you trust the [*provider*] to make a good medical recommendation for your diagnosis? | 1 = not at all; 7 = very much |
|  | 2 | The [*provider*] is concerned about my welfare | 1 = strongly disagree; 7 = strongly agree |
|  | 3 | The [*provider*] provides whatever help I need | 1 = strongly disagree; 7 = strongly agree |
|  | 4 | The [*provider*] acts in my best interest | 1 = strongly disagree; 7 = strongly agree |
|  | 5 | The [*provider*] has good intentions | 1 = strongly disagree; 7 = strongly agree |
|  | 6 | The [*provider*] is competent and effective | 1 = strongly disagree; 7 = strongly agree |
|  | 7 | The [*provider*] performs its role very well | 1 = strongly disagree; 7 = strongly agree |
|  | 8 | The [*provider*] is capable and proficient | 1 = strongly disagree; 7 = strongly agree |
|  | 9 | I’m confident that the medical recommendation from the [*provider*] is accurate | 1 = strongly disagree; 7 = strongly agree |
|  | 10 | I’m confident that the medical recommendation made by the [*provider*] is likely to be reliable | 1 = strongly disagree; 7 = strongly agree |
|  | 11 | I’m confident that the [*provider*] does not fail me | 1 = strongly disagree; 7 = strongly agree |
|  | 12 | I’m confident that the medical recommendation given by the [*provider*] is his/her best judgement | 1 = strongly disagree; 7 = strongly agree |
| Expertise | 1 | The [*provider*] is likely to be highly skilled | 1 = strongly disagree; 7 = strongly agree |
|  | 2 | The [*provider*] knew the latest medical developments | 1 = strongly disagree; 7 = strongly agree |
|  | 3 | I have complete faith in the ability of the [*provider*] | 1 = strongly disagree; 7 = strongly agree |
|  | 4 | Based on what I’ve read, I consider the [*provider*] an expert | 1 = strongly disagree; 7 = strongly agree |
| Attitude | 1 | How would you rate the medical assessment (i.e. the process of how the diagnosis and the medical recommendation were derived)? | 1 = bad; 7 = good  1 = negative; 7 = positive  1= unfavorable; 7 = favorable |
|  | 2 | How does the diagnosis of skin cancer stage [*number*^c^] make you feel? | 1 = bad; 7 = good  1 = negative; 7 = positive  1= unpleasant; 7 = pleasant  1= sad; 7 = happy |
|  | 3 | How does the medical recommendation make you feel? | 1 = bad; 7 = good  1 = negative; 7 = positive  1= unpleasant; 7 = pleasant  1= sad; 7 = happy |
| Treatment Control | 1 | The recommended treatment will be effective in curing my illness | 1 = not at all helpful; 7 = very helpful |
|  | 2 | The negative effects of my diagnosed illness can be prevented by the recommended treatment | 1 = strongly disagree; 7 = strongly agree |
|  | 3 | The recommended treatment can control the diagnosed illness | 1 = strongly disagree; 7 = strongly agree |
|  | 4 | Because of the wide range of positive effects, the recommended treatment is a good way to treat my illness | 1 = strongly disagree; 7 = strongly agree |
|  | 5 | Even if I knew my diagnosis, there is not much I can do about my risk for skin cancer | 1 = strongly disagree; 7 = strongly agree |
| Treatment Risk | 1 | Following the medical recommendation could be harmful | 1 = strongly disagree; 7 = strongly agree |
|  | 2 | Following the medical recommendation could impact my health negatively | 1 = strongly disagree; 7 = strongly agree |
|  | 3 | Following the medical recommendation would cause me too many problems | 1 = strongly disagree; 7 = strongly agree |
|  | 4 | I could make wrong decisions regarding my health based on the poor quality of medical recommendation | 1 = strongly disagree; 7 = strongly agree |
|  | 5 | I would be discouraged to follow the medical recommendation because it would take too much time | 1 = strongly disagree; 7 = strongly agree |
|  | 6 | I would be discouraged to follow the medical recommendation because I would feel silly to do so | 1 = strongly disagree; 7 = strongly agree |
|  | 7 | The medical recommendation involves the risk of an incorrect diagnose | 1 = strongly disagree; 7 = strongly agree |
|  | 8 | The medical recommendation involves the risk of misleading advice | 1 = strongly disagree; 7 = strongly agree |
|  | 9 | The medical recommendation involves the risk that my misinterpretation | 1 = strongly disagree; 7 = strongly agree |
| Treatment Benefit | 1 | I am comfortable providing information to the [*provider*] in return for a treatment recommendation | 1 = strongly disagree; 7 = strongly agree |
|  | 2 | I feel at ease in approaching the [*provider*] to obtain a treatment recommendation | 1 = strongly disagree; 7 = strongly agree |
|  | 3 | Following the medical recommendation gives me greater control over my “health decision” | 1 = strongly disagree; 7 = strongly agree |
|  | 4 | Following the medical recommendation is time-efficient compared to alternatives | 1 = strongly disagree; 7 = strongly agree |
|  | 5 | Following the medical recommendation is cost-efficient compared to alternatives | 1 = strongly disagree; 7 = strongly agree |
|  | 6 | Following the medical recommendation is convenient | 1 = strongly disagree; 7 = strongly agree |
|  | 7 | I am able to follow the medical recommendation without much effort | 1 = strongly disagree; 7 = strongly agree |
|  | 8 | The medical recommendation is tailored to my personal needs | 1 = strongly disagree; 7 = strongly agree |
|  | 9 | The medical recommendation is easily available and accessible | 1 = strongly disagree; 7 = strongly agree |
|  | 10 | The medical recommendation helps me solve my problems | 1 = strongly disagree; 7 = strongly agree |
|  | 11 | The benefits of the medical recommendation outweigh the costs | 1 = strongly disagree; 7 = strongly agree |
| Illness Negative Emotion | 1 | This diagnosed illness worries me | 1 = strongly disagree; 7 = strongly agree |
|  | 2 | This diagnosed illness makes me feel anxious | 1 = strongly disagree; 7 = strongly agree |
|  | 3 | The diagnosed illness makes me feel afraid | 1 = strongly disagree; 7 = strongly agree |
|  | 4 | The thought of skin cancer scares me | 1 = strongly disagree; 7 = strongly agree |
|  | 5 | The thought of developing skin cancer makes me feel scared | 1 = strongly disagree; 7 = strongly agree |
|  | 6 | The fact that so many people have died of skin cancer really frightens me | 1 = strongly disagree; 7 = strongly agree |
| Illness Concern | 1 | How concerned would you be about being diagnosed with skin cancer stage [*number*]? | 1 = not concerned at all; 7 = very concerned |
|  | 2 | How serious would it feel if you were found to have skin cancer stage [*number*]? | 1 = not serious at all; 7 = very serious |
|  | 3 | Skin cancer stage [*number*] is a serious condition | 1 = strongly disagree; 7 = strongly agree |
| Illness Severity | 1 | How would you rate the severity of your symptoms? | 1 = very low; 7 = very high |
|  | 2 | I am certain that if I were to develop skin cancer it would limit my social life | 1 = strongly disagree; 7 = strongly agree |
|  | 3 | If I were to develop skin cancer, I would suffer a lot of pain | 1 = strongly disagree; 7 = strongly agree |
|  | 4 | Developing skin cancer would be unlikely to cause me to die prematurely | 1 = strongly disagree; 7 = strongly agree |
|  | 5 | If I have skin cancer, my life will change | 1 = strongly disagree; 7 = strongly agree |
|  | 6 | If I have skin cancer, I will not be able to do my daily activities | 1 = strongly disagree; 7 = strongly agree |
|  | 7 | Skin cancer can be easily treated | 1 = strongly disagree; 7 = strongly agree |
|  | 8 | I’m afraid of thinking about skin cancer | 1 = strongly disagree; 7 = strongly agree |
|  | 9 | The problems caused by skin cancer remain for a long time | 1 = strongly disagree; 7 = strongly agree |
|  | 10 | If I develop skin cancer it could almost certainly cause my death | 1 = strongly disagree; 7 = strongly agree |
|  | 11 | Skin cancer stage [*number*] has major consequences on my life | 1 = strongly disagree; 7 = strongly agree |
| Illness Vulnerability | 1 | My chance of developing skin cancer in the near future are high | 1 = very unlikely; 7 = very likely |
|  | 2 | I am at risk of getting skin cancer | 1 = very unlikely; 7 = very likely |
|  | 3 | It is likely that I will get skin cancer | 1 = very unlikely; 7 = very likely |
| Privacy Risk | 1 | It would be risky to disclose my personal health information to the [*provider*] | 1 = strongly disagree; 7 = strongly agree |
|  | 2 | There would be high potential for loss associated with disclosing my personal health information to the [*provider*] | 1 = strongly disagree; 7 = strongly agree |
|  | 3 | My personal health information could be inappropriately used by the [*provider*] | 1 = strongly disagree; 7 = strongly agree |
|  | 4 | There would be too much uncertainty associated with giving my personal information to the [*provider*] | 1 = strongly disagree; 7 = strongly agree |
|  | 5 | Providing the [provider] with my personal health information would involve many unexpected problems | 1 = strongly disagree; 7 = strongly agree |
|  | 6 | Compared to others, I am more sensitive about the way my personal health information is handled | 1 = strongly disagree; 7 = strongly agree |
|  | 7 | I believe my personal health information provided to the [*provider*] remains confidential | 1 = strongly disagree; 7 = strongly agree |
|  | 8 | I believe the [*provider*] will prevent unauthorized people from accessing my personal health information in his/her database | 1 = strongly disagree; 7 = strongly agree |
|  | 9 | I believe my personal information is accessible only to those authorized to have access | 1 = strongly disagree; 7 = strongly agree |
| Privacy Concern | 1 | It bothers me when the [*provider*] asks me for this much personal information | 1 = strongly disagree; 7 = strongly agree |
|  | 2 | I am concerned that the [*provider*] is collecting too much personal information about me | 1 = strongly disagree; 7 = strongly agree |
|  | 3 | I am concerned that unauthorized people may access my personal information | 1 = strongly disagree; 7 = strongly agree |
|  | 4 | I am concerned that the [*provider*] may keep my personal information in a non-accurate manner | 1 = strongly disagree; 7 = strongly agree |
|  | 5 | I am concerned about submitting information to the [*provider*] | 1 = strongly disagree; 7 = strongly agree |
| Privacy Control | 1 | I believe I have control over who can get access to my personal health information | 1 = strongly disagree; 7 = strongly agree |
|  | 2 | I think I have control over what personal health information is released by the [*provider*] | 1 = strongly disagree; 7 = strongly agree |
|  | 3 | I believe I have control over how personal health information is used by the [*provider*] | 1 = strongly disagree; 7 = strongly agree |
|  | 4 | I believe I can control my personal health information provided to the [*provider*] | 1 = strongly disagree; 7 = strongly agree |
| Technology Trust | 1 | My typical approach is to trust new technologies until they prove to me that I shouldn’t trust them | 1 = strongly disagree; 7 = strongly agree |
|  | 2 | I usually trust a technology until it gives me a reason not to trust it | 1 = strongly disagree; 7 = strongly agree |
|  | 3 | I generally give a technology the benefit of the doubt when I first use it | 1 = strongly disagree; 7 = strongly agree |
| Technology Readiness | 1 | Technology gives people more control over their daily lives | 1 = strongly disagree; 7 = strongly agree |
|  | 2 | Products and services that use the newest technologies are much more convenient to use | 1 = strongly disagree; 7 = strongly agree |
|  | 3 | You like the idea of doing business via computers because you are not limited to regular business hours | 1 = strongly disagree; 7 = strongly agree |
|  | 4 | You prefer to use the most advanced technology available | 1 = strongly disagree; 7 = strongly agree |
|  | 5 | You like computer programs that allow you to tailor things to fit your own needs | 1 = strongly disagree; 7 = strongly agree |
|  | 6 | Technology gives you more freedom of mobility | 1 = strongly disagree; 7 = strongly agree |
|  | 7 | In general, you are among the first in your circle of friends to acquire new technology when it appears | 1 = strongly disagree; 7 = strongly agree |
|  | 8 | You can usually figure out new high-tech products and services without help from others | 1 = strongly disagree; 7 = strongly agree |
|  | 9 | There should be caution in replacing important people-tasks with technology because new technology can breakdown or get disconnected | 1 = strongly disagree; 7 = strongly agree |
|  | 10 | Many new technologies have health or safety risks that are not discovered until after people have used them | 1 = strongly disagree; 7 = strongly agree |
|  | 11 | New technology makes it too easy for governments and companies to spy on people | 1 = strongly disagree; 7 = strongly agree |
|  | 12 | Technology always seems to fail at the worst possible time | 1 = strongly disagree; 7 = strongly agree |
|  | 13 | People are too dependent on technology to do things for them | 1 = strongly disagree; 7 = strongly agree |
|  | 14 | Too much technology distracts people to a point that is harmful | 1 = strongly disagree; 7 = strongly agree |
|  | 15 | Technology lowers the quality of relationships by reducing personal interaction | 1 = strongly disagree; 7 = strongly agree |
|  | 16 | The human touch is very important when doing business with a company | 1 = strongly disagree; 7 = strongly agree |
|  | 17 | When I call a business, I prefer talking to a person rather than interacting with an automated system | 1 = strongly disagree; 7 = strongly agree |
|  | 18 | Whenever something gets automated, you need to check carefully that the system is not making mistakes | 1 = strongly disagree; 7 = strongly agree |
| Decision Making Preference | 1 | I prefer to make the final selection about which treatment I will receive | 1 = strongly disagree; 7 = strongly agree |
|  | 2 | I prefer to make the final selection of my treatment after seriously considering my [provider]s opinion | 1 = strongly disagree; 7 = strongly agree |
|  | 3 | I prefer that my [*provider*] and I share responsibility for deciding which treatment is best for me | 1 = strongly disagree; 7 = strongly agree |
|  | 4 | I prefer that my [*provider*] makes the final decision about which treatment will be used, but seriously considers my opinion | 1 = strongly disagree; 7 = strongly agree |
|  | 5 | I prefer to leave all decisions regarding my treatment to my [*provider*] | 1 = strongly disagree; 7 = strongly agree |
|  | 6 | I think the decision about my treatment is an emotionally difficult one to make | 1 = strongly disagree; 7 = strongly agree |
|  | 7 | I think the decision about my treatment is stressful | 1 = strongly disagree; 7 = strongly agree |
| Information Preference | 1 | It is important for you to know all the side effects of your medication | 1 = strongly disagree; 7 = strongly agree |
|  | 2 | Information about your illness is as important to you as treatment | 1 = strongly disagree; 7 = strongly agree |
|  | 3 | When there is more than one method to treat a problem, you should be told about each one | 1 = strongly disagree; 7 = strongly agree |
|  | 4 | As you become sicker you should be told more and more about your illness | 1 = strongly disagree; 7 = strongly agree |
|  | 5 | You should understand completely what is happening inside your body as a result of your illness | 1 = strongly disagree; 7 = strongly agree |
|  | 6 | Even if the news is bad, you should be well informed | 1 = strongly disagree; 7 = strongly agree |
|  | 7 | Your [*provider*] should explain the purpose of your laboratory tests | 1 = strongly disagree; 7 = strongly agree |
|  | 8 | You should be given information only when you ask for it | 1 = strongly disagree; 7 = strongly agree |
| Media Exposure | 1 | How much have you heard or read about the use of Big Data or Artificial Intelligence for medical purposes during the last year? | 1 = nothing at all; 7 = very much |
| Perceived Effectiveness | 1 | How effective do you believe Artificial Intelligence is in making better healthcare decision? | 1 = not effective at all; 7 = very effective |
| Age | 1 | Please indicate your age (years): | () |
| Gender | 1 | Please indicate your gender | Male, female, prefer to not answer, other () |
| Education | 1 | Please indicate your highest educational level. | 1 = less than high school diploma, 2 = high school diploma, 3 = some college or associates degree, 4 = bachelor’s degree, 5 = master’s degree, 6 = professional degree, 7 = doctorate |
| History | 1 | Have you or somebody close to you (e.g., spouse, family member) recently been diagnosed with skin cancer | 1 = yes, me, 2 = yes, my spouse/family member, 3 = no |
| Severeness Perception | 1 | How severe do you perceive the diagnosed disease of skin cancer? | 1 = not severe at all; 7 = very severe |
| Incidence Perception | 1 | How would you classify the diagnosed disease of skin cancer? | 1 = every day disease; 7 = once-in-a-lifetime disease |
| Human-Technology Continuum Perception | 1 | Would you rather say that the diagnosis and recommendation was generated by humans or by technology (medical aid system)? | 1 = humans; 7 = technology |

^a^ Depending on the assignment to one of the three experimental conditions, the following terms were used: physician, physician using Artificial Intelligence technology, automated Artificial Intelligence tool. ^b^ Items were dropped to increase the validity and reliability of the scale. ^c^ Depending on the assignment to one of the two experimental conditions, the following levels were mentioned: 0, 2.
